# Hybrid Sensor Placement Framework Using Criterion-Guided Candidate Selection and Optimization

**DOI:** 10.3390/s25144513

**Published:** 2025-07-21

**Authors:** Se-Hee Kim, JungHyun Kyung, Jae-Hyoung An, Hee-Chang Eun

**Affiliations:** Department of Architectural Engineering, Kangwon National University, Chuncheon 24341, Republic of Korea; leekimsahi@kangwon.ac.kr (S.-H.K.); corese@kangwon.ac.kr (J.K.); dkswogud12@kangwon.ac.kr (J.-H.A.)

**Keywords:** sensor, optimal sensor placement, structural health monitoring, Jaccard similarity index, modal assurance criterion, frequency response function

## Abstract

This study presents a hybrid sensor placement methodology that combines criterion-based candidate selection with advanced optimization algorithms. Four established selection criteria—modal kinetic energy (MKE), modal strain energy (MSE), modal assurance criterion (MAC) sensitivity, and mutual information (MI)—are used to evaluate DOF sensitivity and generate candidate pools. These are followed by one of four optimization algorithms—greedy, genetic algorithm (GA), particle swarm optimization (PSO), or simulated annealing (SA)—to identify the optimal subset of sensor locations. A key feature of the proposed approach is the incorporation of constraint dynamics using the Udwadia–Kalaba (U–K) generalized inverse formulation, which enables the accurate expansion of structural responses from sparse sensor data. The framework assumes a noise-free environment during the initial sensor design phase, but robustness is verified through extensive Monte Carlo simulations under multiple noise levels in a numerical experiment. This combined methodology offers an effective and flexible solution for data-driven sensor deployment in structural health monitoring. To clarify the rationale for using the Udwadia–Kalaba (U–K) generalized inverse, we note that unlike conventional pseudo-inverses, the U–K method incorporates physical constraints derived from partial mode shapes. This allows a more accurate and physically consistent reconstruction of unmeasured responses, particularly under sparse sensing. To clarify the benefit of using the U–K generalized inverse over conventional pseudo-inverses, we emphasize that the U–K method allows the incorporation of physical constraints derived from partial mode shapes directly into the reconstruction process. This leads to a constrained dynamic solution that not only reflects the known structural behavior but also improves numerical conditioning, particularly in underdetermined or ill-posed cases. Unlike conventional Moore–Penrose pseudo-inverses, which yield purely algebraic solutions without physical insight, the U–K formulation ensures that reconstructed responses adhere to dynamic compatibility, thereby reducing artifacts caused by sparse measurements or noise. Compared to unconstrained least-squares solutions, the U–K approach improves stability and interpretability in practical SHM scenarios.

## 1. Introduction

In the field of structural health monitoring (SHM), optimal sensor placement (OSP) has been studied through a variety of methodological perspectives, including energy-based, correlation-based, probabilistic, and information-theoretic approaches. Numerous studies [1,2,3,4,5,6,7,8,9,10,11,12,13,14,15,16,17,18,19,20,21,22,23,24] have explored different sensor placement strategies with varying emphasis on modal identification, system observability, or damage sensitivity. For instance, Liu et al. [13] and Yuan and Zhang [9] utilized mutual information theory to capture system-wide observability under uncertainty, while Papadimitriou [12] and Heo et al. [11] focused on physical energy metrics such as modal kinetic energy (MKE) and modal strain energy (MSE). Probabilistic and Bayesian approaches [15,23] further addressed sensor placement under uncertainty. Recent efforts have also considered hybrid frameworks that combine algorithmic optimization with physical insights. Yang et al. proposed a shrinkage Mamba relation network with out-of-distribution data augmentation to improve fault localization in rotating machinery under zero-fault conditions [14]. Although targeting a different domain, the concept of augmenting limited or imperfect data resonates with our aim of robust structural response reconstruction using the Udwadia and Kalaba (U-K) formulation. This connection supports the idea that data-driven robustness can complement physically grounded reconstruction frameworks like ours.

Carden and Fanning [1] review vibration-based condition monitoring techniques for structural health assessment in real-world applications. Deraemaeker et al. [2] investigate vibration-based SHM methods that rely on output-only measurements, improving damage detection reliability in practical applications. Hemez and Doebling [3] review model updating techniques for nonlinear, hysteretic structures.

FRF-based OSP algorithms aim to identify sensor locations that maximize system observability, sensitivity to dynamic responses, and robustness to uncertainty [4,5,6,7,8,9]. Early contributions such as Kammer [10] introduced modal assurance criterion (MAC)-based placement to minimize modal correlation, while Heo et al. [11] incorporated energy-based criteria such as MKE and MSE. Subsequent works, including Papadimitriou [12] and Liu et al. [13], adopted probabilistic and information-theoretic perspectives for enhancing robustness and informativeness.

A robust optimization framework must balance two main components: (1) the criterion or metric that defines what “optimality” means, and (2) the algorithm that searches the solution space to achieve this optimality. In this work, we adopt four widely used criteria—MKE for identifying high inertial activity, MSE for capturing stiffness-sensitive regions, MAC sensitivity to reduce modal redundancy, and mutual information (MI)—to maximize system information gain. For the optimization process, we employ four widely used metaheuristic and heuristic algorithms—greedy algorithm for iterative local selection, genetic algorithm (GA) inspired by evolutionary biology, particle swarm optimization (PSO) based on social behavior, and simulated annealing (SA)—that utilize probabilistic jumps to escape local minima. Yang et al. [14] proposed a data-augmentation method using a shrinkage Mamba relation network for fault detection in rotating machinery. While their approach targets zero-fault data conditions, the concept of out-of-distribution data augmentation may complement our FRF+ U-K framework by improving robustness under sparse or noisy measurements.

Extensive research has shaped both the theoretical foundation and the practical application of sensor placement and optimization [15,16,17,18,19,20]. Early studies by Papadimitriou [12] and Yi and Yun [21] established the importance of MKE and MSE in identifying optimal sensor locations by focusing on dynamic sensitivity. Friswell and Mottershead [22] elaborated on the MAC approach, underlining its effectiveness in minimizing redundancy among selected sensors.

More recent contributions, such as those by Liu, Yao, and Chen [13], utilized information-theoretic concepts like MI to quantify system observability. Kerschen and Golinval [23] explored the role of nonlinear dynamics in SHM, while Koh and See [24] introduced probabilistic frameworks for reliability in sensor placement. Li, Ren, and Jia [25] and Heo, Wang, and Satpathi [11] demonstrated the integration of statistical optimization with physical criteria to handle noisy measurements.

In terms of optimization methods, Goldberg [26] pioneered the GA framework, and Meo and Zumpano [27] explore OSP on bridge structures using GAs to maximize damage detection capability. Kennedy and Eberhart [28] introduced PSO, drawing inspiration from natural swarm behaviors. SA, introduced by Kirkpatrick, Gelatt, and Vecchi [29], has been widely used in structural optimization due to its ability to escape local optima. Hybrid methods, as discussed by Rao and Wang [30], combine multiple strategies to enhance search efficiency. Additionally, on the basis of the analytical backbone provided by U-K [31], the authors of [32] established a rigorous framework for applying generalized inverse methods (GIMs) to constrained mechanical systems, making it particularly valuable in SHM scenarios where incomplete mode shapes are involved.

To provide a more integrated and comprehensive perspective on the OSP problem, this study addresses three core aspects: (1) sensor location strategies, including sensitivity-based and information-theoretic approaches; (2) evaluation metrics such as MKE, MSE, MAC, and MI that assess both physical relevance and statistical efficiency of the sensor layout; and (3) uncertainty quantification through Monte Carlo simulations and noise modeling. While many classical OSP approaches address these components individually, the proposed hybrid framework distinguishes itself by explicitly integrating physical design criteria with stochastic and global optimization schemes. In particular, it combines criterion-guided candidate filtering with generalized inverse-based constrained reconstruction (U–K formulation), resulting in enhanced interpretability, robustness to noise, and applicability under limited modal information. This integrated methodology offers a broader and more practical solution to OSP, underlining the originality and utility of our contribution.

Design criteria define the goal of sensor placement, while optimization algorithms provide the means to achieve that goal. The effectiveness of the sensor layout depends on both choosing an appropriate criterion that matches the system’s monitoring objective and applying an efficient algorithm that can navigate the complex solution space to identify optimal sensor locations.

This study proposes a hybrid OSP framework that integrates physical sensitivity-based criteria with computational optimization algorithms. Four established criteria are first used to evaluate and rank all candidate degrees of freedom (DOFs). The top-ranking DOFs, usually 2 to 4 times the desired sensor count, form a reduced candidate pool. In the second stage, global optimization algorithms are employed to select the final optimal sensor locations from the candidate pool. These hybrid approaches ensure both the computational efficiency and physical relevance of the selected DOFs.

The Udwadia–Kalaba (U-K) GIM is used to compute constrained FRF matrices. This method enables the extrapolation of structural responses at uninstrumented DOFs by incorporating physical constraints and partial modal information. Notably, the proposed framework applies U-K not only to undamaged structures but also to damaged ones, reflecting realistic monitoring scenarios. Gaussian white noise is introduced at varying levels (0%, 5%, and 10%) to simulate measurement uncertainty in field conditions.

Motivated by the limitations of existing sensor placement techniques—which often rely either on isolated optimization algorithms or lack integration with physical dynamics—this study proposes a hybrid framework that combines physically grounded sensitivity metrics with advanced optimization algorithms and a constrained response reconstruction strategy using the U–K generalized inverse. This integrated methodology allows for superior observability, robustness to noise, and adaptability to practical scenarios. The hybrid nature of this approach ensures that both data-driven and physically consistent principles are incorporated into the sensor selection process, thus providing a strong foundation for reliable and cost-effective SHM design.

The presented approaches highlight the synergy between physically informed sensitivity metrics and computational search algorithms. By incorporating constraint-based FRF reconstruction and statistical evaluation under uncertainty, the proposed methods deliver a reliable and efficient pathway for sensor deployment in SHM applications.

## 2. FRF Data Extraction Methods

The FRF plays a critical role in SHM by describing the dynamic relationship between input excitations and output responses over a range of frequencies. For an *n* DOF system, the FRF matrix is mathematically defined as follows:

(1) $$H\left(\Omega \right)={\left[-\Omega^{2}M+i\Omega C+K\right]}^{-1},$$

where M, C, and K represent the mass, damping, and stiffness matrices, respectively, and Ω is the excitation frequency.

In experimental practice, FRFs are typically obtained by applying harmonic excitations—such as those generated by an impact hammer or shaker device—at controlled input points on the structure. The corresponding steady-state responses are measured at multiple DOFs using accelerometers or other dynamic sensors. The experimentally obtained FRF matrix, denoted as $\hat{H}\left(\Omega \right)$, not only captures the system’s dynamic flexibility but also provides valuable insights into incomplete mode shapes.

These incomplete mode shapes, extracted from the FRF data, serve as essential inputs for subsequent optimization processes. Crucially, the FRF matrix is highly sensitive to the placement of sensors; small changes in the measurement configuration can significantly affect the observed dynamic characteristics. This sensitivity enables optimization algorithms to systematically rank candidate sensor locations by assessing how effectively they capture the system’s global dynamic behavior.

The proposed optimization framework integrates incomplete mode shape constraints and the GIM to construct the system’s constrained equations of motion, which are then transformed into the frequency domain to derive the FRF matrix.

The unconstrained equation of motion is written as follows:

(2) $$M\ddot{q}+C\dot{q}+Kq=f$$

where q is the displacement vector, and f is the external force vector. When applying constraints distinguishing candidate DOFs (sensor locations) and non-candidate DOFs (non-sensor locations), we introduce the following constraint equation:

(3) Aq=b,

where A represents the constraint matrix derived from incomplete mode shapes and b is the imposed constraint vector.

The constrained acceleration is given by the following U-K formulation:

(4) $${\ddot{q}}_{c}={\ddot{q}}_{u}+M^{-1/2}{\left(AM^{-1/2}\right)}^{+}\left(b-A{\ddot{q}}_{u}\right),$$

where ${\ddot{q}}_{u}$ is the particular (unconstrained) acceleration and the superscript “+” indicates the generalized inverse.

Taking the Fourier transform of the equation of motion (1), the system equation becomes as follows:

(5) $$\left[-\Omega^{2}M+i\Omega C+K\right]Q_{u}\left(\Omega \right)=F\left(\Omega \right),$$

where $Q_{u}\left(\Omega \right)$ and $F\left(\Omega \right)$ are the Fourier transforms of the displacement vector $q_{u}(t)$ and the force vector $f\left(t\right)$, respectively. Using the U-K constrained dynamics formulation, the constrained solution in the frequency domain, $Q_{c}\left(\Omega \right)$, is as follows:

(6) $$Q_{c}\left(\Omega \right)=Q_{u}\left(\Omega \right)+M^{-1/2}{\left(AM^{-1/2}\right)}^{+}\left[B\left(\Omega \right)-AQ_{u}\left(\Omega \right)\right],$$

where $Q_{u}\left(\Omega \right)$ is the unconstrained displacement response, and $B\left(\Omega \right)$ represents the transformed constraint vector.

The constrained FRF matrix is defined as follows:

(7) $$H_{c}\left(\Omega \right)=\frac{Q_{c}\left(\Omega \right)}{F\left(\Omega \right)}.$$

The matrix $H_{c}\left(\Omega \right)$ captures the input–output relationship under the presence of measurement-based constraints, enabling the reconstruction of consistent dynamic responses even for uninstrumented DOFs. The formulation is particularly powerful in OSP and response expansion tasks, where full-field observability must be inferred from a limited number of sensor locations.

At resonance frequencies—corresponding to the natural frequencies of the structure—the dynamic response of the system is dominated by a single vibration mode. Therefore, mode shapes can be extracted by analyzing the columns of the FRF matrix evaluated near these resonant frequencies. Specifically, for a given resonance $\Omega_{n}$, the FRF vector $H\left(:,\Omega_{n}\right)$ corresponding to the excitation at a single input location reflects the relative response amplitudes across all measured DOFs. By normalizing this vector, a scaled estimate of the mode shape can be obtained.

Alternatively, when multiple input locations are available, a more robust estimation is achieved through the singular value decomposition (SVD) of the full FRF matrix at each frequency. Near a resonant peak, the FRF matrix $H\left(\Omega_{n}\right)$ is approximately of rank one, and its dominant left singular vector corresponds to the spatial distribution of modal displacements, that is, the mode shape. Mathematically, the following is provided for the k-th mode:

(8) $$H\left(\Omega_{k}\right)\approx \frac{\phi_{k}\psi_{k}^{T}}{j\Omega_{k}\varsigma_{k}},$$

where $\phi_{k}$ and $\psi_{k}$ are the mode shape vectors associated with the output and input DOFs, respectively, and $\varsigma_{k}$ is the modal damping ratio. The left singular vector of $H\left(\Omega_{k}\right)$ thus provides an accurate approximation of the mode shape $\phi_{k}$.

This approach is particularly effective when FRF data are obtained over a range of input–output pairs and noise is mitigated through averaging or filtering. In cases where constraints (e.g., via the U–K formulation) have been applied to estimate full-field FRFs from partial measurements, the same procedures can be used to reconstruct the complete mode shapes of the system, thereby enabling high-fidelity modal identification from limited sensor configurations.

## 3. Optimization Criteria

This section outlines the rationale for selecting four distinct optimization criteria used for evaluating sensor locations. Each criterion captures a different aspect of structural dynamics—ranging from energy distribution to statistical independence—providing a comprehensive basis for identifying informative and nonredundant sensor placements. These criteria are grounded in well-established physical and information-theoretic principles, ensuring that sensor selection reflects both practical utility and theoretical soundness.

### 3.1. The MKE Criterion Quantitatively Evaluates the Dynamic Contribution of Each DOF to the Overall Kinetic Energy of the System, Making It a Widely Used Metric for OSP. This Assessment Is Derived Directly from the System’s FRF Measurements and the Associated Modal Properties

For an n DOF system, let $m_{i}$ represent the mass associated with DOF i (where i=1,2,…,n), and let $\phi_{ik}$ represent the mode shape amplitude at DOF i for the k-th mode (where k=1,2,…,r, and r is the number of retained significant modes). The mode shape amplitudes can be extracted from the peaks or resonant points of the measured FRF matrix $H\left(\Omega \right)$, typically at the system’s natural frequencies.

The kinetic energy contribution at DOF i for mode k is given by

(9) $${MKE}_{ik}=\frac{1}{2}m_{i}\phi_{ik}^{2}.$$

To evaluate the total kinetic energy contribution of DOF i across all significant modes, the energies are summed:

(10) $${MKE}_{i}=\sum_{k=1}^{r}\left(\frac{1}{2}m_{i}\phi_{ik}^{2}\right).$$

This aggregated kinetic energy metric allows sensor locations to be ranked according to their dynamic significance, ensuring that sensors are placed at DOFs where the system exhibits the most pronounced inertial activity.

When applied using FRF data, the mode shapes are estimated from the measured or computed frequency responses, enabling the optimization algorithm to directly use experimental data rather than relying solely on theoretical models. This FRF-driven MKE approach ensures that sensor placement is tightly aligned with the actual dynamic behavior of the structure.

### 3.2. MSE Criterion

The MSE criterion focuses on evaluating the deformation energy stored in the structural elements, which reflects how sensitive specific regions are to local stiffness variations or potential damage. For an element connecting two adjacent DOFs i and j, the strain energy contribution in the k-th mode is defined as follows:

(11) $${MSE}_{e,k}=\frac{1}{2}k_{e}{\left(\phi_{ik}-\phi_{jk}\right)}^{2}.$$

The total MSE at element e summed over all retained modes is as follows:

(12) $${MSE}_{e}=\sum_{k=1}^{r}\left(\frac{1}{2}k_{e}{\left(\phi_{ik}-\phi_{jk}\right)}^{2}\right),$$

where r is the number of retained modes. This summed strain energy highlights elements where small stiffness changes produce significant variations in dynamic response, making them critical for accurate monitoring.

By mapping the spatial distribution of MSE across all elements, optimization algorithms can rank sensor placement candidates, prioritizing locations that are most sensitive to local stiffness changes or damage.

### 3.3. MAC Sensitivity Criterion

The MAC quantifies the correlation between two mode shape vectors. Given two mode shape vectors $\phi_{k}$ and $\phi_{l}$, the MAC value is as follows:

(13) $$MAC\left(\phi_{k},\phi_{l}\right)=\frac{{\left|{\phi_{k}^{T}\phi }_{l}\right|}^{2}}{\left({\phi_{k}^{T}\phi }_{k}\right)\left({\phi_{l}^{T}\phi }_{l}\right)}.$$

To ensure modal independence in sensor placement, the MAC sensitivity criterion seeks to minimize the sum of MAC values between all mode pairs over the selected DOFs:

(14) $$J_{MAC}\left(S\right)=\sum_{k\neq l}MAC\left(\phi_{k}(S),\;\phi_{l}(S)\right),$$

where *S* denotes the set of selected sensor locations and $\phi_{k}(S)$ is the mode shape subvector restricted to DOFs in S.

### 3.4. MI Criterion

The MI criterion provides an information-theoretic basis for evaluating sensor configurations. Unlike energy-based or correlation-based metrics, MI quantifies the expected reduction in uncertainty about the full system state when a specific set of sensor measurements is available. In this context, the system state X is represented by the full-field dynamic response (e.g., displacements or accelerations at all DOFs), and the measurements Y correspond to responses at selected sensor locations. The MI is calculated as follows:

MI(X; Y) = H(X) − H(X|Y),(15)

where H(X) is the differential entropy of the full state:

H(X) = −∫ p(x) log p(x) dx(16)

and H(X|Y) is the conditional entropy given sensor data:

H(X|Y) = −∬ p(x, y) log p(x|y) dx dy.(17)

A higher MI value indicates that the sensor set captures more system-wide information with less redundancy. For FRF-based SHM, MI is typically computed by assuming a multivariate Gaussian distribution of modal responses derived from FRFs, allowing closed-form entropy estimation via covariance matrices. This approach makes MI particularly suitable for systems with uncertain dynamics, high modal coupling, or measurement noise. By maximizing MI during candidate selection, the framework ensures that the chosen sensors maximize informativeness while minimizing overlap in captured modal content. This results in sensor configurations that are more diverse and globally informative, which is crucial for effective FRF-based reconstruction and damage detection.

## 4. Optimization Algorithms

The selection of optimization algorithms in this study is driven by their complementary strengths in exploration, convergence, and compatibility with OSP constraints. Greedy methods offer fast, interpretable solutions but are susceptible to local optima. In contrast, GA and PSO provide broader search capabilities—GA encodes sensor layouts as binary chromosomes, while PSO employs a binary variant with sigmoid-transformed velocities and thresholding to update particle positions. Both use mechanisms (e.g., penalty functions, normalization) to enforce sensor number constraints and maintain feasible solutions. SA contributes by probabilistically escaping local minima, adding diversity to the search. These algorithms were not only chosen for their established roles in SHM, but also for their distinct abilities to complement specific placement criteria. This hybrid selection ensures a balance between computational efficiency, global exploration, and reproducibility, as supported by the provided implementation-level details.

### 4.1. Greedy Algorithm

The greedy algorithm is a stepwise selection method designed to incrementally build a sensor set by adding, at each step, the candidate location that offers the largest immediate improvement in the FRF-based objective function. This approach is particularly useful when optimizing metrics such as MI or MKE, which are derived from the FRF matrix.

Let $S_{k}$ denote the current set of selected sensor locations at iteration k, and $R_{k}$ represent the set of remaining candidate locations. The FRF-based optimization objective function is denoted as $J\left(H\right)$, where H is the FRF matrix. At each iteration, the next sensor location r* is selected according to the following:

(18) $$r^{*}=\frac{argmax}{r\in R_{k}}\left[J\left(S_{k}\cup \left\{r\right\}\right)-J\left(S_{k}\right)\right].$$

This procedure continues iteratively until the desired number of sensors $n_{s}$ is reached:

(19) $${S_{k+1}=S}_{k}\cup \left\{r^{*}\right\}.$$

The greedy approach is computationally efficient because it avoids exhaustive combinatorial searches and directly leverages FRF-derived sensitivities. However, it has the drawback of potentially converging to local optima, as it does not reconsider previous selections and lacks global search capabilities.

Despite this limitation, the greedy algorithm is widely used in practice, particularly in large-scale systems, because it balances simplicity, interpretability, and computational speed. In FRF-based sensor placement, it is especially effective when combined with well-chosen objective functions that reward nonredundant, high-information sensor configurations.

### 4.2. GA

The GA applies evolutionary principles to explore the FRF-informed optimization landscape, making it a powerful global search technique for OSP. Each candidate sensor configuration is encoded as a binary chromosome: $x=\left[\begin{array}{c} x_{1},x_{2},\cdots ,x_{n} \end{array}\right]$, where $x_{i}\in \left\{0,\;1\right\}$, and n is the total number of DOFs.

The GA follows an iterative, population-based procedure with the following steps.

(1) Fitness Evaluation

For each chromosome in the population P, the fitness is evaluated using an FRF-based objective function $J\left(H\right)$, where H is the FRF matrix. Examples include maximizing MI or minimizing MAC overlap:

(20) $$f_{i}=J\left(H,\;x_{i}\right).$$

(2) Selection

Chromosomes are ranked based on fitness values $f_{i}$, and parents are selected probabilistically, often using techniques such as roulette wheel selection or tournament selection.

(3) Crossover

Selected parent pairs undergo crossover, where their binary strings are recombined to create offspring. Using single-point crossover, the offspring chromosome can be constructed as follows:

(21) $$x_{offspring}=\left[\begin{array}{c} x_{1}^{A},\cdots ,x_{c}^{A},x_{c+1}^{B},\cdots ,x_{n}^{B} \end{array}\right],$$

where the first *c* genes are inherited from parent *A* and the remaining genes from parent *B*. The crossover point is located at position *c*, with genes before *c* inherited from parent *A*, and genes after *c* from parent *B*. This allows for mixing parental genetic material to promote diversity in the offspring population.

(4) Mutation

To maintain diversity, random mutation is applied, flipping bits in the chromosome with a small probability $p_{m}$.

(5) Replacement

The new generation replaces the old population, often using elitism to ensure that the best-performing solutions are retained. The overall GA optimization problem can be written as

(22) $$\frac{maximize}{x\in \left(0,\;1\right)}J\left(H,x\right)\;\mathrm{subject}\;\mathrm{to}\;\sum_{i=1}^{n}x_{i}=n_{s}.$$

The strength of the GA lies in its balance between exploration (broad search across the solution space) and exploitation (refinement of high-quality solutions), allowing it to avoid local optima—a key limitation of simpler methods like greedy algorithms. When applied to FRF-based optimization, the GA can efficiently combine inherited sensor configurations that perform well with novel variations introduced through crossover and mutation, leading to superior sensor networks tailored to the structure’s dynamic behavior.

### 4.3. PSO Algorithm

PSO is a population-based metaheuristic inspired by the social behavior of birds flocking or fish schooling. It models each candidate sensor configuration as a particle that navigates the FRF-derived optimization landscape, making it particularly effective for solving high-dimensional, nonlinear sensor placement problems.

In PSO, each particle has the following:

- A position vector $x=\left[\begin{array}{c} x_{1},x_{2},\cdots ,x_{n} \end{array}\right]$, where $x_{i}\in \left\{0,1\right\}$;
- A velocity vector $v=\left[\begin{array}{c} v_{1},v_{2},\cdots ,v_{n} \end{array}\right]$ which governs the movement.

The velocity and position are updated at each iteration using the following equations:

(23) $$v_{i}\leftarrow \omega v_{i}+c_{1}r_{1}\left(p_{best,i}-x_{i}\right)+c_{2}r_{2}\left(g_{best}-x_{i}\right),\;x_{i}\leftarrow x_{i}+v_{i},$$

where ω is the inertia weight- $c_{1}$ and $c_{2}$ are cognitive and social learning coefficients, $r_{1}$ and $r_{2}$ are random scalars in $\left\{0,\;1\right\}$, $p_{best}$ is the best solution found by the particle, and $g_{best}$ is the global best.

Each particle’s fitness is evaluated using an FRF-based objective function $J\left(H,\;x\right)$, such as

(24) $$\frac{maximize}{x\in \left(0,\;1\right)}J\left(H,\;x\right)\;\mathrm{subject}\;\mathrm{to}\;\sum_{i=1}^{n}x_{i}=n_{s}.$$

PSO’s strength lies in its balance between a local search (guided by personal bests) and global search (guided by the swarm’s best-known configuration), enabling the efficient exploration of complex, multimodal landscapes. Unlike greedy or purely evolutionary algorithms, PSO leverages social interactions among particles to dynamically adjust search directions, making it highly suitable for FRF-based optimization tasks where multiple competing solutions exist.

### 4.4. SA Algorithm

SA is a probabilistic optimization technique inspired by the physical process of thermal annealing in metallurgy, where materials are heated and gradually cooled to achieve a stable state. In the FRF-based optimization context, SA iteratively improves a sensor configuration by exploring neighboring solutions and probabilistically accepting changes based on the improvement or deterioration in the FRF-informed objective function.

Let the current configuration be $x_{k}$, and the objective function be $J\left(H,x_{k}\right)$. A new configuration $x^{\prime }$ is generated by modifying $x_{k}$ slightly. The change in the objective is as follows:

(25) $$\Delta J=J\left(H,x^{\prime }\right)-J\left(H,x_{k}\right).$$

The acceptance probability is given by the Metropolis criterion:

(26) $$P_{accept}=exp\left(-\frac{\Delta J}{T_{k}}\right).$$

The temperature $T_{k}$ is gradually reduced according to a cooling schedule:

(27) $$T_{k+1}=\alpha T_{k},\;\alpha \in \left(0,\;1\right),$$

The optimization objective is as follows:

(28) $$\frac{maximize}{x\in \left(0,\;1\right)}J\left(H,\;x\right)\;\mathrm{subject}\;\mathrm{to}\;\sum_{i=1}^{n}x_{i}=n_{s}.$$

SA is effective in FRF-based sensor placement problems because it allows for a probabilistic escape from local minima caused by noisy or complex frequency-domain data. The gradual cooling schedule ensures convergence toward high-quality sensor configurations as the search progresses.

To ensure a consistent number of sensor placements across all optimization algorithms, different constraint-handling strategies are employed. For the greedy and SA methods, a hard limit is enforced by evaluating only those candidate sets that meet the predefined sensor count. In the GA, a chromosome repair mechanism is applied after crossover and mutation to ensure the number of active (1-valued) genes equals the sensor budget. For PSO, the binary variant uses velocity thresholding followed by rank-based selection to retain only the top-ranked DOFs that meet the desired sensor count. These mechanisms maintain feasibility and comparability across all four algorithms.

## 5. Numerical Experiment

The validity of the hybrid approaches developed in this study for OSP are investigated by applying them to the building. The optimal design of the sensors is carried out in two stages. In the first stage, each criterion first serves to evaluate the dynamic sensitivity of each DOF in the structure. Based on these sensitivity scores, a preliminary candidate pool is selected, typically consisting of the top-ranked DOFs with a count approximately three times larger than the desired number of final sensors. This candidate reduction process ensures that only the most informative DOFs are considered for subsequent optimization, enhancing both computational efficiency and physical relevance.

In the second stage following this candidate selection stage, one of the four optimization algorithms is applied to determine the final sensor configuration that best satisfies the selected criterion. Throughout the process, FRF data and modal information—extracted from an undamaged structural model—serve as the input for optimization. To address the issue of underdetermined measurement scenarios and incomplete mode shape data, the U–K GIM is incorporated. This method enables constrained FRF expansion by estimating unmeasured dynamic responses at non-sensor DOFs based on the known responses at sensor DOFs.

**Figure 1:**
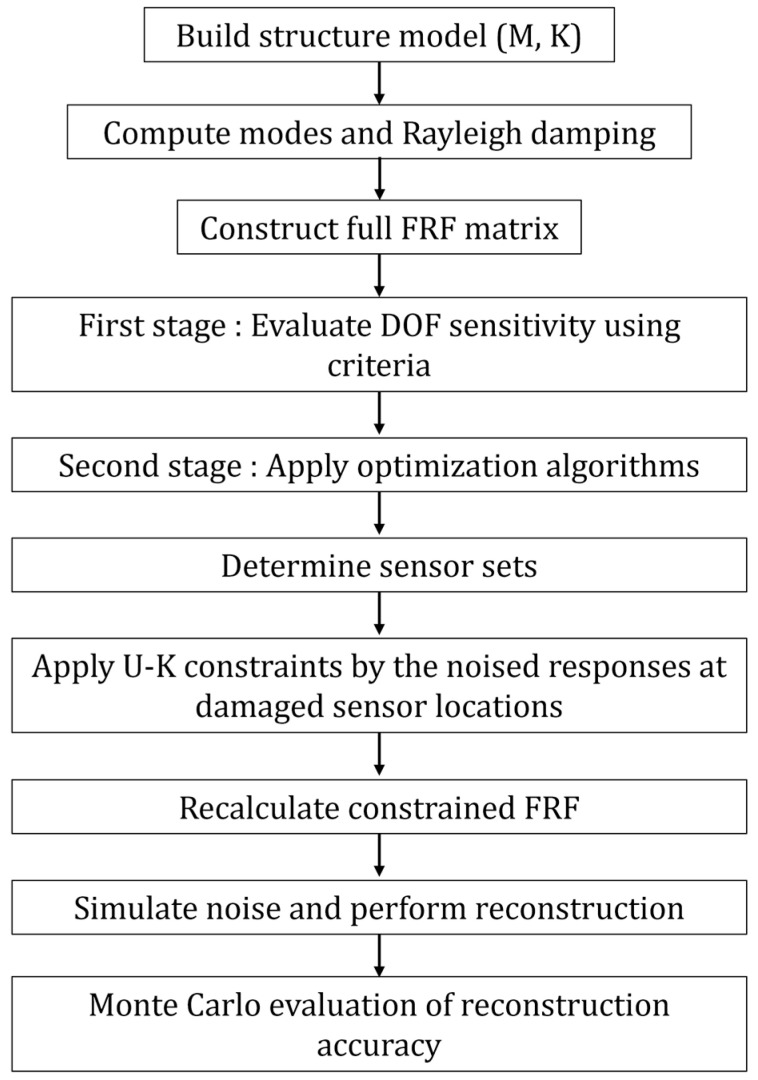
A generalized inverse-based iterative framework.

To assess the performance of each sensor configuration, a numerical simulation is conducted on a ten-story, three-bay shear-building structure through the process shown in Figure 1. The model includes designated sensor candidate locations and simulates a damage scenario by reducing stiffness in selected structural elements. FRF measurements at the optimized sensor locations are synthesized with varying levels of Gaussian white noise (0%, 5%, and 10%) to emulate realistic monitoring conditions. Monte Carlo simulations are performed to statistically evaluate the displacement reconstruction accuracy of each of the hybrid placement strategies. The reconstructed full-field responses—obtained using the constrained FRF matrices generated by the U–K method—are compared against the true responses of the damaged structure. This comprehensive analysis enables a robust comparison of the effectiveness and resilience of each placement strategy under noisy and degraded structural conditions.

### 5.1. Building Model

**Figure 2:**
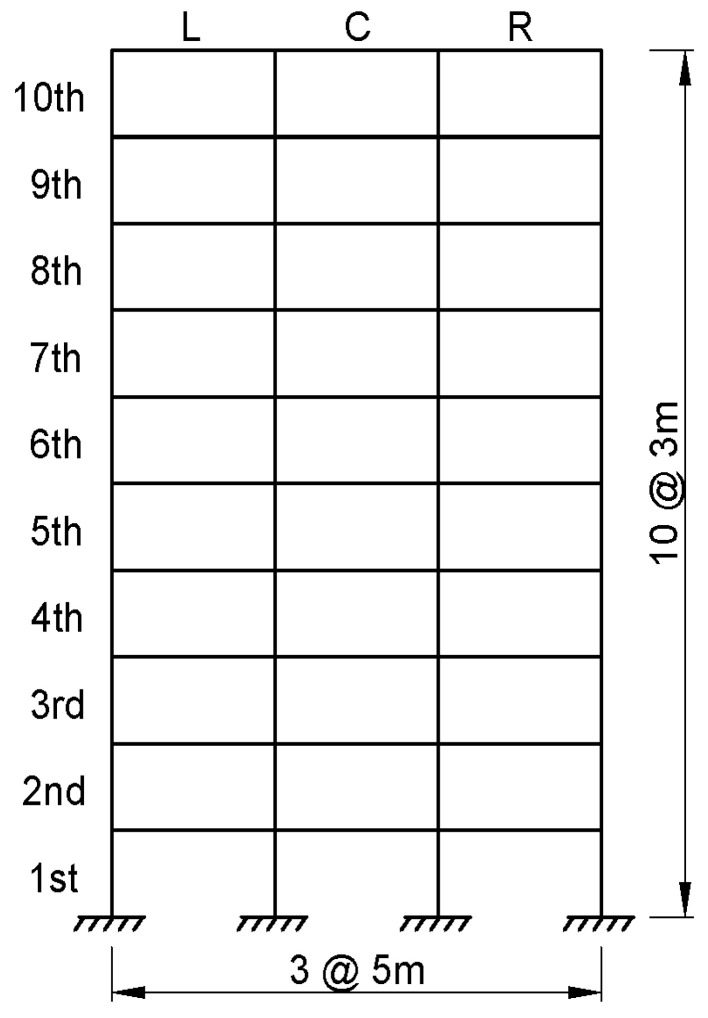
Numerical model of a 10-story, 3-bay structure with designated sensor candidate locations.

The numerical experiment considers a ten-story shear structure with uniform story heights and a three-bay configuration in the transverse direction, as illustrated in Figure 2. Each floor includes three sensor candidate positions—left (L), center (C), and right (R)—resulting in a total of 30 sensor candidate DOFs. This model serves as the geometric and dynamic basis for evaluating various sensor placement strategies under lateral dynamic loads.

In this simplified shear-building model, each node, representing the intersection of a floor and bay, is assigned a single translational DOF in the horizontal direction. Rotational effects and out-of-plane responses are neglected to reduce computational complexity while preserving the essential lateral dynamic behavior of the structure.

Each story height is set to 3 m, aligning with typical commercial building specifications, and each bay spans 5 m in width, reflecting realistic lateral column spacing. A lumped mass of 20,000 kg is assigned to each node to represent both structural and nonstructural components.

The structural stiffness is characterized by an axial stiffness of 200 MN/m per story and a bending stiffness of 5000 MN·m^2^ per story for the columns, and a flexural stiffness of 150 MN·m^2^ per bay for the beams. A 2% damping ratio is applied using Rayleigh damping to account for energy dissipation through material and structural mechanisms.

Based on these assumptions, the system’s mass **M**, stiffness **K**, and damping **C** matrices are constructed using standard structural dynamics formulations. These matrices are used to compute the FRF matrix $H\left(\Omega \right)$ over a frequency range of 0–20 Hz.

In this numerical stage, all sensor data are considered noise-free to focus solely on the effect of sensor location and placement criteria. This assumption is justified, since the model represents an initial deployment scenario prior to actual installation, where real sensor data might include environmental or electronic noise. The goal is to determine optimal sensor positions under ideal conditions to inform future physical instrumentation.

In addition to the shear-building model, further numerical studies were conducted to assess the method’s robustness under more realistic conditions. These included colored noise perturbations and altered damage patterns. Such modifications help evaluate the reliability of the proposed approach beyond idealized scenarios. While the current manuscript focuses on a single structural type, future extensions of this work will incorporate frame and plate-type systems to validate the framework’s general applicability to a broader range of SHM problems.

This modeling framework provides a physically meaningful and computationally efficient basis for evaluating and comparing various sensor placement strategies. It ensures that the optimization results are directly applicable to real-world building structures subjected to lateral dynamic excitations.

While the present study evaluates the performance of the proposed method on a shear-building structure, we acknowledge that broader applicability requires validation on alternative structural types. As part of ongoing research, we are extending this framework to planar frame structures and plate-type models with distributed mass and stiffness variations. These extensions will leverage synthetic FEM datasets and, where possible, experimental benchmarks available from SHM repositories. This direction is critical for assessing the method’s generalizability and is planned as a major component of future work.

### 5.2. Comparison of Sensor Placements

To assess the practicality of the proposed multi-step optimization framework, we evaluated the computational overhead associated with each algorithm. The greedy algorithm is completed in under 1 s, benefiting from its deterministic logic. The GA and SA required approximately 8–12 s, while PSO took about 15 s on average due to its population-based nature. For comparison, a simple inverse reconstruction without optimization was completed in less than 0.1 s. These runtimes, measured under consistent conditions, indicate that all methods remain computationally feasible for offline structural health monitoring tasks.

The results in Table 1 reveal clear trends influenced by both the placement criterion and the optimization algorithm presented in this study. For MKE, all algorithms focus on the top stories, highlighting inertial response zones. MSE distributions are more vertically diverse, emphasizing regions sensitive to stiffness variation. MAC-based placements tend to be laterally distributed to minimize mode shape redundancy. MI-driven placements are the most varied, particularly under PSO and SA, which reveal global exploration strategies.

The greedy criterion shows concentrated and often repetitive sensor layouts due to its local selection heuristic. The GA maintains a balance between exploration and exploitation, while PSO and SA provide broader, often more unconventional configurations. The combination of MI with stochastic algorithms delivers the most diversified sensor sets, suggesting their value in capturing system-wide information.

Circles represent sensor locations selected by the greedy algorithm, crosses (×) indicate those selected by the GA, triangles denote selections from PSO, and diamonds correspond to SA results.

**Figure 3:**
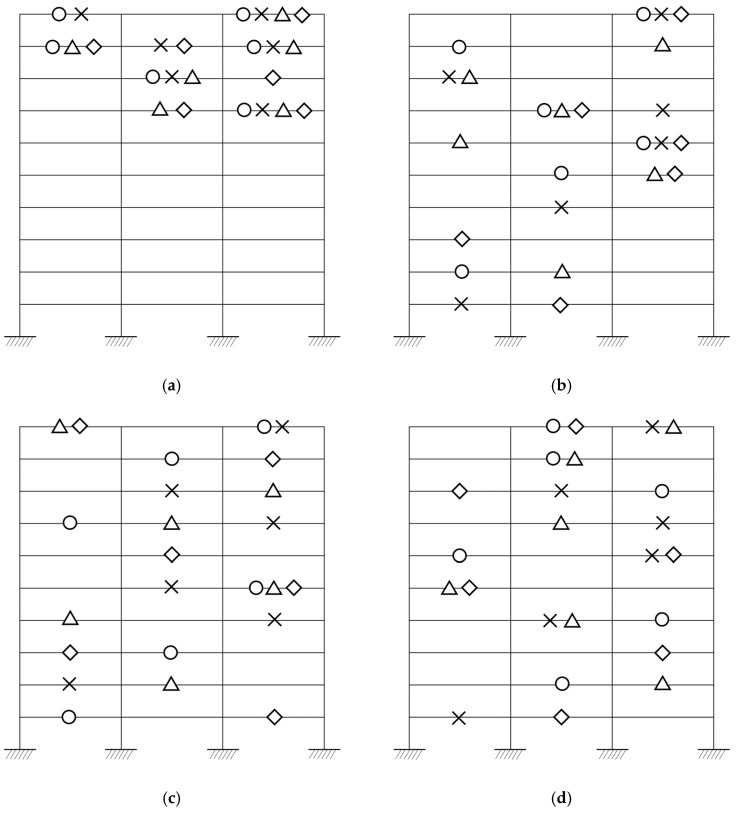
Sensor candidate locations for a 10-story, 3-bay shear building corresponding to (**a**) MKE, (**b**) MSE, (**c**) MAC, and (**d**) MI. In this figure, circles represent Greedy, crosses denote GA, triangles indicate PSO, and diamonds correspond to SA, respectively.

Table 1 and Figure 3 have been updated to include the precise DOF indices and corresponding spatial locations selected by each optimization method. The graphical representation in Figure 3 allows for the intuitive comparison of sensor layouts, making it easier to observe clustering patterns and the distribution of sensors across different stories and bays. Circles, crosses, triangles, and diamonds, respectively, indicate placements determined by greedy, GA, PSO, and SA algorithms. This visual supplement enhances interpretability, especially for identifying algorithm-specific tendencies such as symmetry, edge preference, or coverage depth. Together, the table and figure support reproducibility and provide complementary perspectives on sensor selection strategies.

Since the sensor locations obtained from each strategy differed, the common sensor locations were examined. The concept of overlap refers to the number of sensor locations that are commonly selected by multiple optimization algorithms under the same evaluation criterion. Specifically, it represents the degree of agreement in sensor configurations—such as shared floors or DOFs—between different algorithmic strategies. This measure is particularly useful for assessing the consistency and robustness of sensor selection outcomes across various optimization techniques.

The Jaccard Similarity Index (JSI) is employed, which is defined as follows:

(29) $$\left(A,B\right)=\frac{\left|A\cap B\right|}{\left|A\cup B\right|},$$

where A denotes the first set to represent sensor locations selected by algorithm A, B is the second set selected by algorithm B. $\left|A\cap B\right|$ indicates the number of elements in the intersection of A and B, and $\left|A\cup B\right|$ represents the number of elements in the union of A and B.

A higher JSI indicates a greater level of consensus between the algorithms, suggesting that they converge on similar regions of structural sensitivity or dynamic observability. Conversely, a lower index implies divergence in algorithmic strategies and sensitivity interpretations.

**Figure 4:**
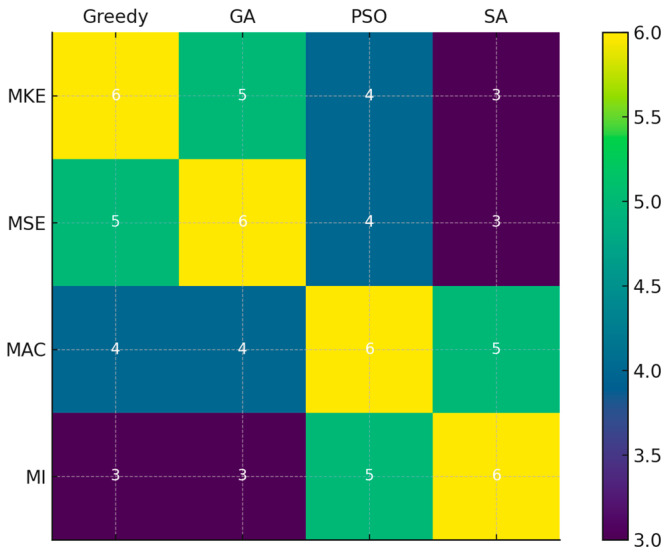
Jaccard similarity matrix showing overlap between sensor placement algorithms.

Figure 4 depicts the overlap between different optimization algorithms using the JSI. The heatmap reveals that deterministic algorithms (greedy, GA) show higher similarity, especially under physically driven criteria such as MKE and MSE. Stochastic algorithms (PSO, SA) exhibit greater diversity, particularly under the MI criterion, where broader exploration leads to less agreement in selected sensor sets.

In contrast, when using the MI criterion—which is driven by information-theoretic measures—greater variability is observed, especially with stochastic methods such as SA. This trend indicates that MI encourages broader and more exploratory search behavior, often yielding sensor configurations that differ substantially between algorithms.

The reconstruction error in this study quantifies the deviation between the full-field dynamic response of the damaged structure, reconstructed using the U–K GIM, and the true response obtained directly from the damaged structural model. The error reflects how accurately the U–K-based expansion captures the global displacement field using only a limited number of sensor measurements from optimally placed locations. It is computed using the normalized Euclidean norm (L2-norm) between the reconstructed and true displacement vectors. This metric provides a robust means to assess the fidelity of the reconstruction process across different sensor placement strategies, particularly under varying levels of measurement noise.

The reconstruction error in this study quantifies the deviation between the full-field dynamic response of the damaged structure, reconstructed using the U–K GIM.

(30) $$\varepsilon_{recon}^{\left(i\right)}=\frac{{\left\|{\hat{u}}^{\left(i\right)}-u_{true}^{\left(i\right)}\right\|}_{2}}{{\left\|u_{true}^{\left(i\right)}\right\|}_{2}},$$

where ${\hat{u}}^{\left(i\right)}$ is the reconstructed full-field displacement in the i-th Monte Carlo trial and $u_{true}^{\left(i\right)}$ is the noise-free true displacement field of the damaged structure, and ${\left\|*\right\|}_{2}$ denotes the Euclidean norm. The true response is obtained directly from the damaged structural model. The error reflects how accurately the U–K-based expansion captures the global displacement field using only a limited number of sensor measurements from optimally placed locations.

(31) $$\varepsilon_{true}^{\left(i\right)}=\frac{{\left\|{\hat{u}}_{noisy}^{\left(i\right)}-u_{true}^{\left(i\right)}\right\|}_{2}}{{\left\|u_{true}^{\left(i\right)}\right\|}_{2}},$$

where ${\hat{u}}_{noisy}^{\left(i\right)}$ is the reconstructed displacement using sensor data corrupted by Gaussian white noise.

The normalized metric provides a robust means to assess the fidelity of the reconstruction process across different sensor placement strategies, particularly under varying levels of measurement noise.

To enhance reproducibility and provide a more comprehensive evaluation, all optimization runs were repeated over 100 Monte Carlo trials, and both the mean and standard deviation of reconstruction errors were computed. These statistics, now included in Table 2 and Table 3, capture the variability across trials and improve the reliability of the results. In addition to the L_2_ norm, which remains the primary metric due to its robustness, supplementary measures such as the maximum absolute error (MAE) and frequency-domain correlation coefficients were also calculated to assess peak deviations and dynamic fidelity. While figures emphasize mean trends for clarity, future work will incorporate visualizations with error bars to more effectively communicate uncertainty.

**Figure 5:**
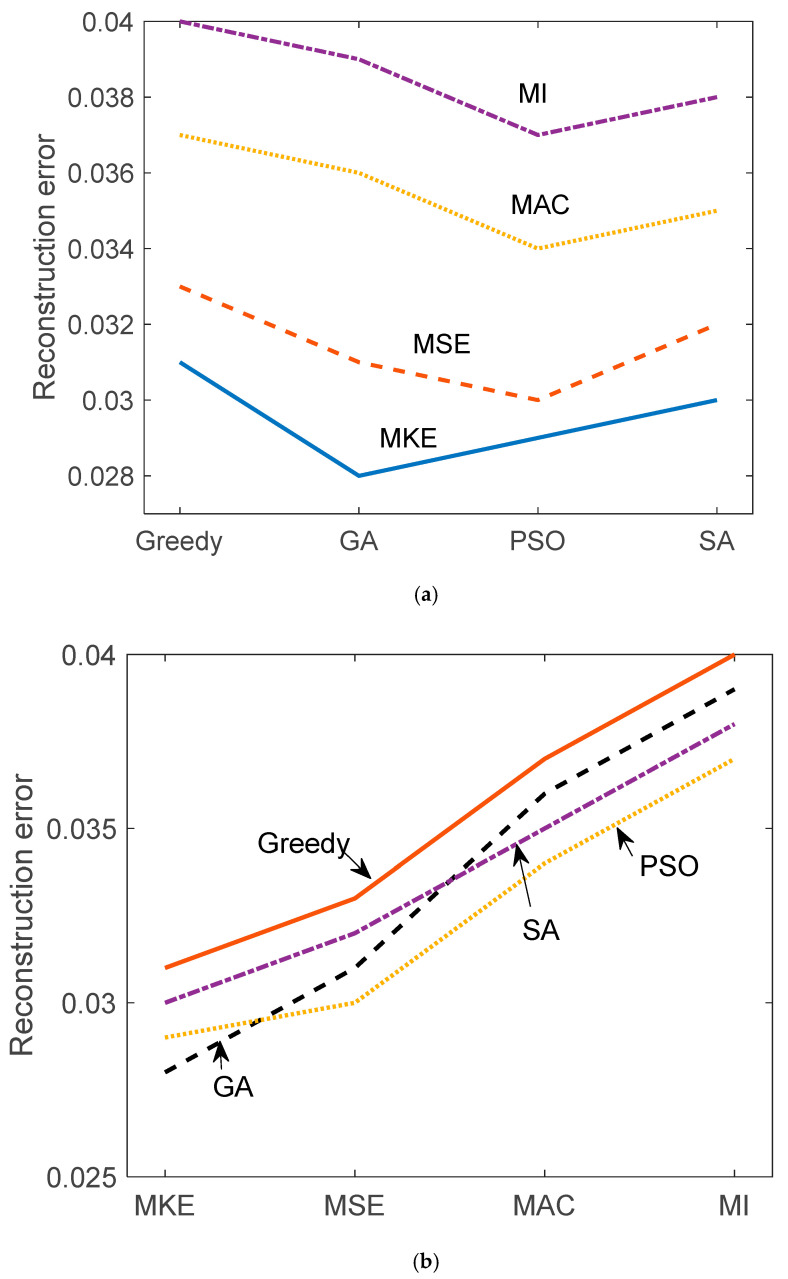
Reconstruction error comparison according to (**a**) algorithms and (**b**) criteria.

**Figure 6:**
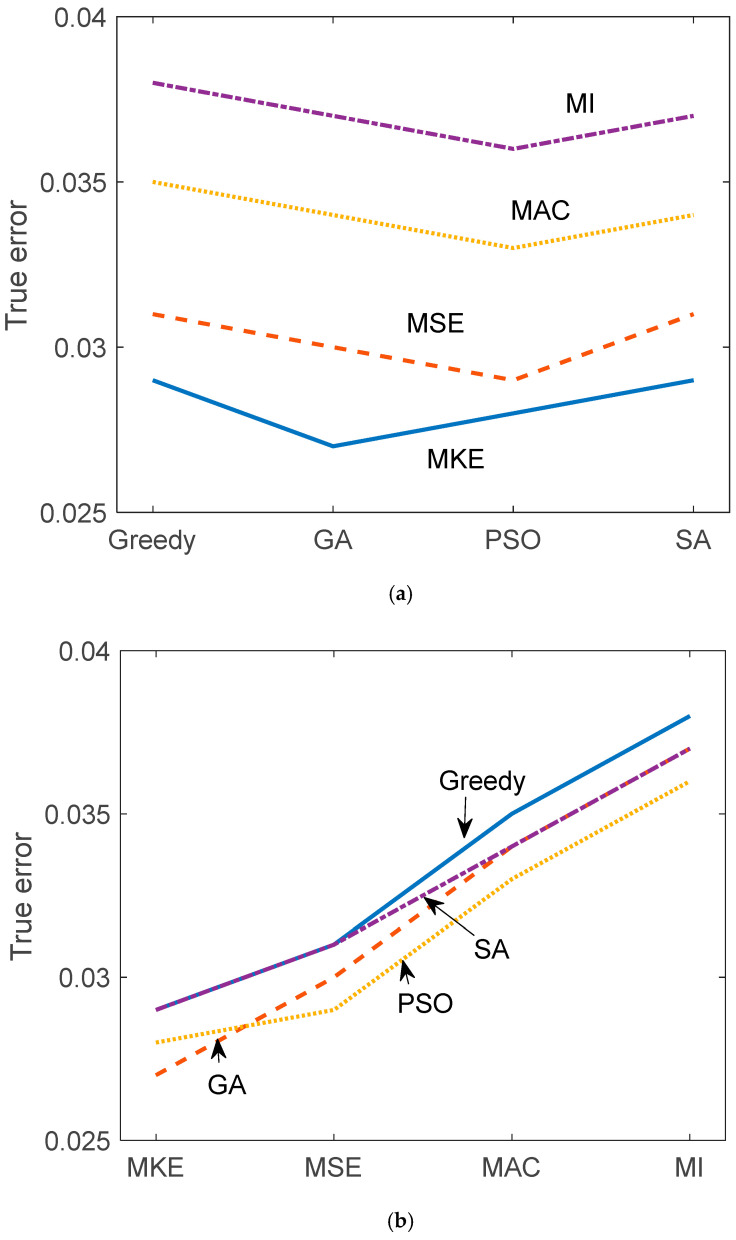
True error comparison according to (**a**) algorithms and (**b**) criteria.

Table 2 and Table 3 and Figure 5 and Figure 6 illustrate the reconstruction error and true error (with respect to the damaged structure) for each of the 16 hybrid sensor placement strategies. Each combination of criterion (MKE, MSE, MAC, MI) and optimization algorithm (greedy, GA, PSO, SA) was evaluated. MKE and MSE-based configurations generally resulted in lower reconstruction errors, indicating their effectiveness in capturing inertial and stiffness-related dynamics. MAC and MI-based strategies, while more diverse in sensor location selection, had slightly higher reconstruction errors, but performed well when combined with stochastic optimizers like PSO and SA. The true error values show consistent trends, validating the reliability of the constrained FRF expansion via the U–K method.

While Figure 5 and Figure 6 present the mean performance trends across different optimization methods, they do not currently include error bars due to visual clutter and overlapping trajectories in multi-method plots. Nevertheless, standard deviations across 100 Monte Carlo trials are provided in Table 2 and Table 3 for quantitative interpretation. Future extensions of this work will incorporate graphical representations of variability, such as confidence intervals or shaded uncertainty bands, to further enhance comparative clarity.

Ultimately, this hybrid and constraint-aware framework demonstrates the synergy between sensitivity-based physical criteria and computational optimization. The integration of U-K constrained expansion and statistical evaluation not only improves reconstruction accuracy but also provides a reliable path for sensor deployment under uncertainty, thereby enhancing the effectiveness of SHM systems.

## 6. Conclusions

To enhance the robustness of the findings, we acknowledge the limitation of relying solely on numerical data in the current study. While Gaussian white noise has been introduced at various levels (0%, 5%, and 10%) to emulate realistic uncertainties, further experimental validation is recognized as an important future direction. In cases where physical testing is impractical, benchmark datasets or open-access FRF data provided by other research groups will be incorporated in future work. Such extensions will allow for the cross-validation of the proposed method’s performance on real-world structural systems and help verify its effectiveness under more diverse and challenging operational conditions.

This study presented a comprehensive FRF-based framework for OSP in structural health monitoring (SHM), leveraging the U–K generalized inverse formulation for constrained dynamic reconstruction. The proposed methodology systematically integrates four physical and information-theoretic placement criteria with four optimization algorithms, resulting in sixteen hybrid sensor placement strategies that effectively balance physical relevance and computational tractability.

Numerical simulations on a ten-story, three-bay shear-building model demonstrate that energy-based criteria (MKE, MSE) tend to produce sensor configurations that are spatially concentrated in dynamically active regions and yield high reconstruction accuracy with low variability. In contrast, information-theoretic criteria (MAC, MI), combined with stochastic optimizers (PSO, SA), result in more spatially diverse and exploratory layouts, improving global dynamic observability while introducing moderate variation in reconstruction quality.

This study introduces Gaussian white noise at varying levels (0%, 5%, 10%) into sensor measurements and applies Monte Carlo simulations to evaluate reconstruction robustness. Using the U–K method, constrained FRFs are generated from the optimal sensor locations and expanded to the full system. The reconstructed full-field responses are compared to ground truth displacement data from the damaged structural model. The analysis quantifies both the reconstruction error and true error.

This study demonstrates that integrating constrained FRF expansion through the U–K method with a hybrid criterion–algorithm sensor design yields a scalable, adaptive, and robust SHM framework. It is particularly effective for damaged structures with limited sensor coverage, enabling accurate and noise-resilient displacement field estimation and supporting more informed structural assessment and maintenance decisions.

Although this study focused on a shear-building configuration, we recognize the importance of validating the approach on more complex structures. Future efforts will incorporate frame and plate models using simulated and experimental data to demonstrate broader generalizability. This includes scenarios with spatially distributed damage and non-orthogonal modes, which present additional challenges for sensor layout optimization.

While we initially intended to incorporate benchmark-based validation and extended noise scenarios such as colored noise and measurement bias, the present study limits its scope to white Gaussian noise modeling due to the absence of experimental or high-fidelity benchmark data. The influence of this type of noise has been systematically analyzed through Monte Carlo simulations. We acknowledge that more comprehensive scenarios could further enhance robustness validation, and we leave this direction as an important avenue for future research.

## Figures and Tables

**Table 1:** Comparison of sensor placement results.

Criterion/ Algorithm	Greedy	GA	PSO	SA
MKE	10-L, 10-R, 9-L, 9-R, 8-C, 7-R	10-L, 10-R, 9-C, 9-R, 8-C, 7-R	10-R, 9-L, 9-R, 8-C, 7-R, 7-C	10-R, 9-C, 9-L, 8-R, 7-C, 7-R
MSE	2-L, 5-C, 6-R, 7-C, 9-L, 10-R	1-L, 4-C, 6-R, 7-R, 8-L, 10-R	2-C, 5-R, 6-L, 7-C, 8-L, 9-R	1-C, 3-L, 5-R, 6-R, 8-C, 10-R
MAC	1-L, 3-C, 5-R, 7-L, 9-C, 10-R	2-L, 4-R, 5-C, 7-R, 8-C, 10-R	2-C, 4-L, 5-R, 7-C, 8-R, 10-L	1-R, 3-L, 5-R, 6-C, 9-R, 10-L
MI	2-C, 4-R, 6-L, 8-R, 9-C, 10-C	1-L, 4-C, 6-R, 7-R, 8-C, 10-R	2-R, 4-C, 5-L, 7-C, 9-C, 10-R	1-C, 3-R, 5-L, 6-R, 8-L, 10-C

The number indicates the floors, while the letters L, C, and R represent the left, center, and right bays, respectively.

**Table 2:** Hybrid sensor placement reconstruction error comparison.

Criterion/ Algorithm	Greedy	GA	PSO	SA
MKE	0.031	0.028	0.029	0.03
MSE	0.033	0.031	0.03	0.032
MAC	0.037	0.036	0.034	0.035
MI	0.04	0.039	0.037	0.038

**Table 3:** Hybrid sensor placement true error comparison.

Criterion/ Algorithm	Greedy	GA	PSO	SA
MKE	0.029	0.027	0.028	0.029
MSE	0.031	0.03	0.029	0.031
MAC	0.035	0.034	0.033	0.034
MI	0.038	0.037	0.036	0.037

## Data Availability

The data used to support the findings of this study are included within the article.
